# miR-23a/b suppress cGAS-mediated innate and autoimmunity

**DOI:** 10.1038/s41423-021-00668-x

**Published:** 2021-03-25

**Authors:** Qiuya Yu, Lei Chu, Yongxing Li, Quanyi Wang, Juanjuan Zhu, Chen Wang, Shufang Cui

**Affiliations:** State Key Laboratory of Natural Medicines, School of Life Science and Technology, China Pharmaceutical University, Nanjing, China

**Keywords:** cGAS, miR-23a, miR-23b, DNA virus, Innate immunity, Autoimmune disease, Innate immunity, Autoimmunity

## Abstract

Cyclic GMP-AMP synthase (cGAS), a key sensor of intracellular DNA, is essential for eliciting innate immunity against infection, whereas aberrant activation of cGAS by endogenous DNA promotes severe autoimmune diseases. However, it is largely unknown how cGAS expression is regulated during pathogen infection and autoimmunity. Here, we report that during herpes simplex virus type 1 (HSV-1) infection, two microRNAs (miR-23a and miR-23b) whose levels significantly decrease due to their interaction with the lncRNA Oasl2-209 directly regulate the expression of cGAS. Overexpression of miR-23a/b markedly dampens cytosolic DNA-induced innate immune responses, whereas inhibition of miR-23a/b enhances these responses. Mice treated with miR-23a/b agomirs exhibit increased susceptibility to HSV-1 infection. Moreover, cGAS is significantly upregulated in the *Trex1*^*−/−*^ mouse autoimmune disease model. Administration of miR-23a/b blunts self DNA-induced autoinflammatory responses in *Trex1*^*−/−*^ mice. Collectively, our study not only reveals a novel regulatory mechanism of cGAS expression by miRNAs but also identifies a potential therapy for cGAS-related autoimmune diseases.

## Introduction

The innate immune system provides a first line of defense against pathogen invasion. Recognition of pathogen-associated molecular patterns through germline-encoded receptors initiates the induction of type I interferons (IFNs) that mediate innate immune responses.^1^ Innate immune DNA sensing is crucial for host defense and constitutes a general mechanism to detect the presence of various pathogens.^2^ Cyclic GMP-AMP (cGAMP) synthase (cGAS) has recently been defined as a key sensor of cytosolic DNA and is responsible for IFN-mediated innate immune signaling.^3^ Interaction with double-stranded DNA (dsDNA) triggers cGAS activation and production of the second messenger 2'3'-cGAMP.^4,5^ cGAMP then binds to the endoplasmic reticulum protein stimulator of interferon genes (STING),^6^ which in turn results in the activation of the downstream effectors TANK-binding kinase 1 (TBK1) and interferon regulatory factor 3 (IRF3), ultimately inducing the expression of type I IFNs and interferon-stimulated genes (ISGs).^7^

Although cGAS indispensable for pathogen defense, its aberrant activation by endogenous DNA is a key driver of autoimmune diseases. Loss-of-function mutations in *TREX1*, a DNA 3' repair exonuclease that degrades cytosolic DNA, have been identified in autoimmune disorders such as Aicardi–Goutières syndrome (AGS) and familial chilblain lupus in human patients.^8,9^ *Trex1*^*−/−*^ mice exhibit autoimmune responses similar to those of AGS patients and are a tractable model in which to study AGS diseases.^10,11^ It has been demonstrated that chronic activation of cGAS by accumulated cytoplasmic self DNA causes autoimmune diseases in *Trex1*^*−/−*^ mice and that genetic ablation of *Cgas* rescues all the pathological and molecular phenotypes.^11–13^ These studies confirm the importance of cGAS inhibition in the treatment of self DNA-induced autoimmune diseases.

Given the critical role of cGAS in microbial infection defense and autoimmune diseases, it is urgent to understand the regulation of cGAS activation and expression. Several types of posttranslational modifications have been found to modulate cGAS activity.^14–16^ However, little is known about the regulation of cGAS expression to date, and the potential mechanisms deserve further exploration. MicroRNAs (miRNAs) are well-known posttranscriptional regulators of gene expression via binding to the 3' untranslated regions (3'-UTRs) of target mRNAs to induce their degradation or suppress their translation.^17,18^ It has been demonstrated that miRNAs play critical roles in various biological processes, including innate immunity, inflammation and autoimmune diseases.^19,20^ miR-23a and miR-23b (miR-23a/b) belong to the miR-23–27–24 family and differ by just one nucleotide in the nonseed region. The functions of miR-23a/b have been widely described in cancer and T cell differentiation^21–23^ but have rarely been linked to innate immunity until recently.^24–27^ Time course experiments have shown that miR-23a expression decreases at early time points in herpes simplex virus type 1 (HSV-1)-infected cells, facilitating HSV-1 replication through the suppression of interferon regulatory factor 1 in human HeLa cells.^28^ In addition, miR-23a/b are downregulated in human inflammatory lesions such as those of rheumatoid arthritis and inhibit IL-17-associated autoimmune inflammation by targeting IKKα.^24,25^ Although evidence suggests the important roles of miR-23a/b in viral infection and autoimmune inflammation, relevant knowledge about whether they can function as regulators of IFN-β production and related autoimmunity remains limited. Interestingly, bioinformatic analysis predicted a binding site for the miR-23a/b seed sequences in the 3'-UTR of cGAS, revealing the possibility that miR-23a/b could be involved in the cGAS-mediated pathway.

In this study, we identified cGAS as a direct target of miR-23a/b via their interaction with the 3'-UTR of cGAS mRNA. Decreased miR-23a/b levels contributed to an increase in the cGAS protein level in HSV-1-infected cells. Overexpression of miR-23a/b suppressed cGAS-mediated innate immune responses, whereas inhibition of miR-23a/b enhanced these responses. Accordingly, mice treated with miR-23a/b agomirs were more susceptible to HSV-1 infection. Moreover, our data indicated an inverse change trend between the levels of miR-23a/b and the cGAS protein level in *Trex1*^*−/−*^ mice. Injection of miR-23a/b agomirs ameliorated the disease phenotype of *Trex1*^*−/−*^ mice, providing evidence that miR-23a/b might be a new therapeutic target for autoimmune diseases.

## Materials and methods

### Mice and treatment

*Trex1*^+/−^ mice on a C57BL/6 background were kindly licensed by Dr. Tomas Lindahl and Dr. Deborah Barnes (Cancer Research UK) and provided by Dr. Nan Yan (University of Texas Southwestern Medical Center). These mice were intercrossed to generate wild-type (WT) and *Trex1*^*−/−*^ mice, which were genotyped by standard PCR. All mice were maintained under specific pathogen-free conditions at the Center for New Drug Safety Evaluation and Research, China Pharmaceutical University.

For in vivo viral infection studies, 8-week-old WT male mice (purchased from the Model Animal Research Center of Nanjing University) were injected intravenously (i.v.) with miR-23a/b agomirs or control agomirs (1000 nmol/kg) (RiBoBio, Guangzhou, China). Forty-eight hours after injection, the mice were infected (i.v.) with HSV-1 at a dose of 5 × 10^7^ pfu per mouse for 12 h. For in vivo autoimmune response studies, 4-week-old *Trex1*^*−/−*^ mice were injected intraperitoneally (i.p.) with miR-23a/b agomirs or control agomirs (500 nmol/kg) for 6 days. Animal experiments were carried out in accordance with the National Institutes of Health Guide for the Care and Use of Laboratory Animals.

### Cell culture and transfection

HEK293 (originally purchased from ATCC), MEF (originally purchased from ATCC), and HeLa (originally purchased from ATCC) cells were cultured in DMEM (Invitrogen) containing 10% fetal bovine serum (Gibco) and 1% penicillin-streptomycin (Invitrogen). L929 cells (originally purchased from ATCC) were cultured in RPMI-1640 (Gibco) with the same supplements. BMDMs were generated as previously described.^29^ Cells were maintained in a humidified incubator at 37 °C with 5% CO_2_. Transfection was performed with Lipofectamine 3000 (Invitrogen) according to the manufacturer’s instructions.

### Antibodies and reagents

The antibody against TBK1 (ab40676) was purchased from Abcam. Antibodies against cGAS (#31659), IRF3 (#4302), phospho-IRF3 (#4947), and phospho-TBK1 (#5483) were purchased from Cell Signaling Technology. The anti-GAPDH (sc-32233) antibody was purchased from Santa Cruz Biotechnology. Anti-MB21D1 (HPA031700) and anti-β-actin (A5441) antibodies were purchased from Sigma-Aldrich. The cyanine 3-conjugated secondary antibody (goat anti-rabbit) was purchased from Jackson ImmunoResearch.

Herring testis DNA (HT-DNA) was purchased from Sigma-Aldrich. HSV-1 and HSV-1-GFP were kindly provided by Dr. Wentao Qiao (Nankai University) and Dr. Chunfu Zheng (Suzhou University), respectively. HSV-1 was propagated and titered by plaque assays in Vero cells.

### Plasmids and siRNAs

The coding sequence of cGAS and cDNA of Oasl2-209 were obtained using standard PCR techniques and subsequently cloned into the pcDNA3.1 vector. The empty pcDNA3.1 vector was used as a negative control. To construct the cGAS-WT reporter plasmid, a 290-bp fragment of the cGAS 3'-UTR containing the conserved miR-23a/b binding site was inserted into the pmirGLO vector (Promega). To test binding specificity, the sequence that interacted with the miR-23a/b seed sequence was mutated from ATGTGA to TACACT to construct the mutant vector (cGAS-MUT). Similarly, a 250-bp fragment of Oasl2-209 containing the miR-23a/b binding site was inserted into the pmirGLO vector to construct the Oasl2-209-WT reporter plasmid, and the sequence that interacted with the miR-23a/b seed sequence was then mutated from GTGTGGT to TACACCA to construct the Oasl2-209-MUT plasmid.

siRNA duplexes targeting Oasl2-209 were chemically synthesized by GenePharma. The siRNA sequence was as follows: si-Oasl2-209, 5'-GGA AGA CUG AUG ACA UUA UTT-3'.

### miRNA overexpression and knockdown

Synthetic miRNA mimics and inhibitors and their corresponding negative control oligonucleotides were purchased from RiBoBio (Guangzhou, China). For miRNA overexpression and knockdown, cells were seeded in 12-well plates or 6-well plates. Then, 60 nM miRNA mimics or 100 nM miRNA inhibitors and their corresponding negative controls were added to cells using Lipofectamine 3000 (Invitrogen). Forty-eight hours after transfection, cells were harvested for total RNA or protein extraction.

### RNA isolation and quantitative PCR (qPCR)

Total RNA was extracted from cultured cells and tissues with TRIzol reagent (Invitrogen) according to the manufacturer’s instructions. Stem-loop primers specific for miRNAs were used to perform reverse transcription with a HiScript III 1st Strand cDNA Synthesis Kit (+gDNA Wiper) (Vazyme). qPCR was performed using ChamQ SYBR qPCR Master Mix (Low ROX Premixed) (Vazyme), and U6 was used as the endogenous reference. For quantification of mRNA, the extracted RNA was reverse transcribed using HiScript III Q RT SuperMix (Vazyme) for qPCR, and mouse *Gapdh* was used as the internal control. The comparative Ct method (2^−△△Ct^) was used to calculate the relative expression levels of targets. The primer sequences used for all genes are shown in Supplementary Table 1.

### Immunoblot analysis

Extracts from cultured cells were prepared in lysis buffer (50 mM Tris-HCl (pH 7.4), 150 mM NaCl, 1% Triton X-100, 1 mM EDTA) supplemented with complete protease inhibitor cocktail (Sigma) for 30 min at 4 °C. Extracts from tissues were prepared in RIPA lysis buffer (Beyotime). Then, the lysates were separated by SDS-PAGE and electrophoretically transferred to a PVDF membrane (Millipore). The membrane was probed first with the indicated primary antibodies overnight at 4 °C and then with horseradish peroxidase-conjugated secondary antibodies for 1 h, and immunoreactions were then visualized by using a SuperSignal West Pico Chemiluminescence ECL kit (Pierce). The band intensities on the blots were quantified with ImageJ software.

### IRF3 dimerization assay

Cell extracts were prepared in native lysis buffer (50 mM Tris-HCl (pH 8.0), 150 mM NaCl, 1% NP40, 1% protease inhibitor cocktail, and 1% orthovanadate) for 30 min at 4 °C. Lysates were loaded onto a native PAGE gel (without SDS), which was prerun in electrophoresis buffer (25 mM Tris-HCl (pH 8.4) and 192 mM glycine with and without 0.2% deoxycholate in the cathode and anode chamber, respectively) for 60 min at 40 mA and electrophoresed for 60 min at 25 mA. Then, the native gel was soaked in SDS electrophoresis buffer (25 mM Tris (pH 8.3), 250 mM glycine, and 0.1% SDS) for 30 min at room temperature and analyzed by immunoblotting as described above.

### RNA-seq experiments and analysis

RNA-seq was performed by Novogene Company (Beijing). Total RNA was extracted from control and HT-DNA-stimulated L929 cells using TRIzol reagent (Invitrogen) and converted into a cDNA library according to the standard Illumina RNA-seq instructions. High-throughput sequencing was performed in an Illumina NovaSeq 6000 system by paired-end 150 bp sequencing. Clean reads were mapped to the mouse reference genome (Ensemble_GRCm38.90) with Hisat2 software (version 2.0.5). Quantification of transcripts and genes was performed using StringTie software (version 1.3.3).

### Measurement of IFN-β

The concentration of IFN-β in culture supernatants was measured with a mouse IFN-beta ELISA kit (4A Biotech) according to the manufacturer’s instructions.

### Measurement of cGAMP

Cell and tissue extracts were prepared in M-PERTM Extraction Reagent (Thermo Fisher Scientific). After centrifugation for 20 min, the supernatants were heated at 95 °C for 5 min to remove denatured proteins. The concentration of cGAMP in the supernatants was measured with a 2'3'-cGAMP ELISA kit (Cayman Chemical) according to the manufacturer’s instructions.

### Luciferase reporter assay

Cells were seeded in 24-well plates. Then, 50 ng of the constructed reporter plasmids was cotransfected into cells with 60 nM miRNA mimics, 100 nM miRNA inhibitors or their corresponding negative control RNAs using Lipofectamine 3000 (Invitrogen). After the cells were cultured for 24 h, firefly and Renilla luciferase activities were assayed using a Dual-Luciferase Reporter Assay System (Promega) according to the manufacturer’s instructions. The relative luciferase activities were calculated based on the ratio of firefly/Renilla fluorescence.

### Pulldown assay with biotinylated miRNA

Cells were transfected with 80 nM biotinylated miR-23a/b mimics (miR-23a/b probes) or control probe (RiBoBio, Guangzhou, China) and were then harvested in lysis buffer (20 mM Tris-HCl (pH 7.5), 100 mM KCl, 5 mM MgCl_2_, 0.5% NP40 and 200 U/ml RNase inhibitor (Vazyme)). The lysate was next incubated with streptavidin magnetic beads (Invitrogen) at 4 °C overnight with constant rotation. After incubation, the beads were washed with lysis buffer four times, and RNA was extracted with TRIzol (Invitrogen) for analysis.

### Pulldown assay with the biotinylated DNA probe

The 3'-terminal biotinylated probe complementary to the long noncoding RNA (lncRNA) Oasl2-209 sequence was synthesized, and a scrambled biotinylated probe was used as the negative control (Genescript, Nanjing, China). These probes (10 pmol/µl) were then incubated with streptavidin magnetic beads (Invitrogen) for 2 h with constant rotation. Cells were harvested in lysis buffer (20 mM Tris-HCl (pH 7.5), 100 mM KCl, 5 mM MgCl_2_, 0.5% NP40, and 200 U/ml RNase inhibitor (Vazyme)). Then, the lysates were incubated with the probe-coated beads at 4 °C overnight with constant rotation. After incubation, the beads were washed with wash buffer (10 mM Tris-HCl, pH 7.5, 1 mM EDTA, and 2 M NaCl) three times, and RNA was extracted with TRIzol (Invitrogen) for analysis. The sequences of the probes were as follows: Oasl2-209 probe, 5'-CCC AAA TTA TCA TTA CCA CC-3'; control probe, 5'-TGA TGT CTA GCG CTT GGG CTT TG-3'.

### Immunofluorescence

To detect the localization of IRF3, L929 cells were seeded on glass coverslips in 12-well plates and transfected with miRNA mimics or inhibitors. After treatment with HT-DNA, cells were fixed with 4% paraformaldehyde (Beyotime) in PBS for 15 min, permeabilized with 0.25% Triton X-100 for 15 min, and blocked in 5% BSA for 1 h. Cells were then incubated with the indicated primary antibodies overnight at 4 °C and with cyanine 3-conjugated secondary antibodies for 1 h. Nuclei were stained with DAPI (Sigma). Images of the cells were acquired using a confocal microscope (LSM700, Carl Zeiss) with a ×63 oil immersion objective.

### Flow cytometry

Cells seeded in 6-well plates were transfected with miR-23a/b mimics or inhibitors for 48 h and were then infected with HSV-1-GFP (MOI = 0.3) for 16 h. The ratio of GFP-positive cells to total cells was detected by flow cytometry (Invitrogen Attune NxT Flow Cytometer).

### Histological analysis

Tissues were fixed with 4% paraformaldehyde (Beyotime), embedded in paraffin, sliced into sections, and placed on adhesive microscope slides. Sections were stained with hematoxylin and eosin (H&E) for pathological analysis. Cardiac inflammation was scored on H&E sections as previously described.^30^ For immunohistochemical (IHC) staining, the anti-cGAS antibody (Cell Signaling Technology) was used.

### Statistical analysis

All data were analyzed using Prism 5 software (GraphPad Software, Inc.). Data are expressed as the mean ± standard deviation (SD) values. Two-tailed Student’s *t*-test was performed on a minimum of *n* = 3 replicates for statistical analysis. Differences with a *p* value < 0.05 were considered significant.

## Results

### miR-23a/b directly regulate cGAS expression

**Fig. 1:**
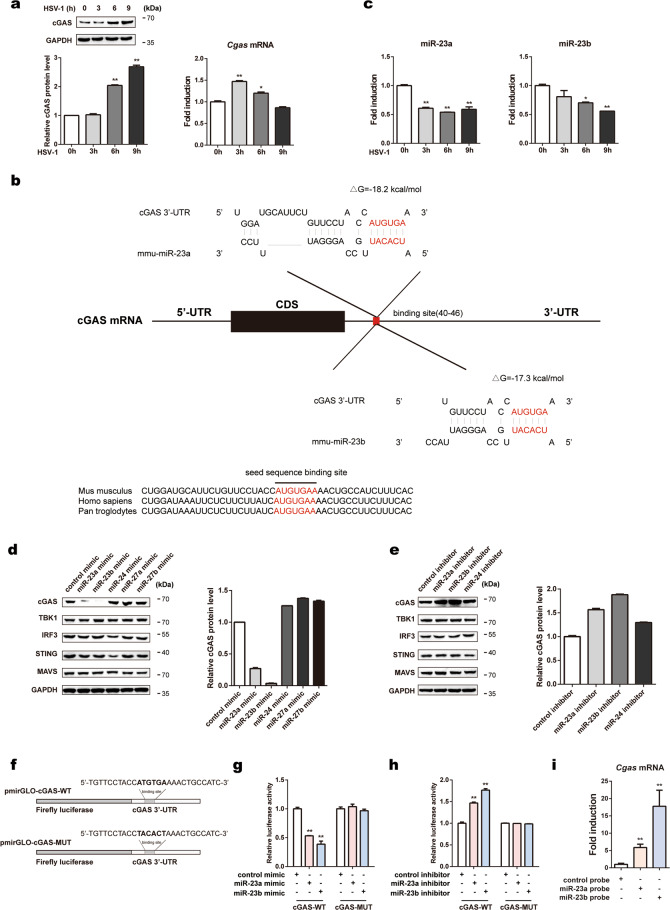
miR-23a/b directly regulate cGAS expression. **a** L929 cells were infected with HSV-1 (MOI = 1) for the indicated times. Then, the cGAS protein level was analyzed by western blotting, and the relative band intensity was quantified by ImageJ (left panel). The cGAS mRNA level was measured by qPCR (right panel). **b** Schematic description of the predicted interactions between miR-23a/b and their binding site in the cGAS 3'-UTR. The binding site and miR-23a/b seed sequences are marked in red, and the predicted minimum free energy values of the hybridizations are shown. **c** L929 cells were infected with HSV-1 (MOI = 1) for the indicated times. The levels of miR-23a/b were then measured by qPCR. L929 cells were transfected with the indicated mimics (**d**) or inhibitors (**e**), and the protein levels of cGAS, TBK1, IRF3, STING, and MAVS were then analyzed by western blotting. The relative band intensity of cGAS was quantified by ImageJ. **f** Fragments of the cGAS 3'-UTR were cloned into pmirGLO reporter plasmids. The mutated nucleotides are marked in bold. HEK293 cells were transfected with miR-23a/b mimics (**g**) or inhibitors (**h**) along with the indicated reporter plasmids for 24 h. Then, the relative luciferase activities were analyzed. **i** MEFs were transfected with biotinylated miR-23a/b probes for 48 h and were then harvested for a biotin-avidin pulldown assay. RNA was extracted, and the cGAS mRNA levels were measured by qPCR. The data are representative of three independent experiments (mean ± SD). *p < 0.05, **p < 0.01

To investigate the regulatory mechanism of cGAS expression in innate immune responses against viral infection, we first infected L929 cells and MEFs with the DNA virus HSV-1. The cGAS protein level increased persistently and markedly after HSV-1 infection in both cell lines, whereas the cGAS mRNA level increased marginally at early time points (3 h for L929 cells and 4 h for MEFs) and exhibited a decreasing trend thereafter (Fig. 1a and Supplementary Fig. 1a). Given the inconsistency between the protein and mRNA levels of cGAS, we suspected that the protein expression of cGAS could be regulated by miRNAs. As shown by the TargetScan database, the 3'-UTR of cGAS contains a conserved miR-23a/b binding site (Fig. 1b). The RNAhybrid program showed that the minimum free energy values of the two predicted hybridizations were −18.2 and −17.3 kcal/mol, well within the range of genuine miRNA-target pairs. Subsequently, we examined the expression of miR-23a/b during HSV-1 infection. The levels of miR-23a/b in both L929 cells and MEFs were significantly decreased, revealing a change trend opposite that observed for cGAS protein expression after HSV-1 infection (Fig. 1c and Supplementary Fig. 1b). By contrast, this pattern was not observed for miR-24, miR-27a, or miR-27b, the other members of the miR-23–27–24 family (Supplementary Fig. 1b, c).

To investigate whether miR-23a/b can regulate the expression of the cGAS protein, we overexpressed or knocked down miR-23a/b by transfecting L929 cells with miR-23a/b mimics or inhibitors. Upregulation of miR-23a/b dramatically decreased the cGAS protein level, whereas downregulation of miR-23a/b increased the cGAS protein level (Fig. 1d, e). However, both miR-23a and miR-23b barely influenced the cGAS mRNA level (Supplementary Fig. 1d, e), indicating that miR-23a/b negatively regulate cGAS expression at the posttranscriptional level. Notably, the protein levels of STING, TBK1, IRF3, and MAVS (mitochondrial antiviral signaling protein), all of which are essential for type I IFN signaling responses, were barely affected by miR-23a/b modulation (Fig. 1d, e). In addition, transfection of miR-24/27a/27b mimics or inhibitors had little effect on the cGAS protein level (Fig. 1d, e). Importantly, consistent results were also observed in MEFs and human HeLa cells (Supplementary Fig. 1f, g). Taken together, these results validate the specific modulation of the cGAS protein level by miR-23a/b.

To further study whether the negative regulation of cGAS expression is mediated through binding of miR-23a/b to the predicted site in the cGAS 3'-UTR, we cloned the cGAS 3'-UTR harboring the wild-type (cGAS-WT) or mutant (cGAS-MUT) miR-23a/b target sequence into pmirGLO plasmids (Fig. 1f). The cGAS-WT or cGAS-MUT reporter plasmids were transfected into HEK293 cells along with miR-23a/b mimics, inhibitors, or the corresponding negative control RNAs. Overexpression of miR-23a/b suppressed luciferase reporter activity (Fig. 1g), whereas inhibition of miR-23a/b resulted in the opposite effect (Fig. 1h). By contrast, neither overexpression nor inhibition of miR-23a/b had an effect on the luciferase activity of the mutant reporter (Fig. 1g, h). Furthermore, we performed a biotin-avidin pulldown assay to test whether miR-23a/b can directly bind to cGAS mRNA. MEFs were transfected with miR-23a/b mimics with biotinylated 3'-termini (miR-23a/b probes) and were then harvested for a biotin-based pulldown assay, and the coprecipitated cGAS mRNA was analyzed by qPCR. cGAS mRNA was greatly enriched in the immunoprecipitates pulled down with the miR-23a and miR-23b probes compared to those pulled down with the control probe (Fig. 1i), confirming that miR-23a/b can directly bind to cGAS mRNA. Collectively, these results demonstrate that miR-23a/b directly regulates cGAS expression by targeting the 3'-UTR of cGAS.

### LncRNA Oasl2-209 directly binds to miR-23a/b and regulates miR-23a/b expression

**Fig. 2:**
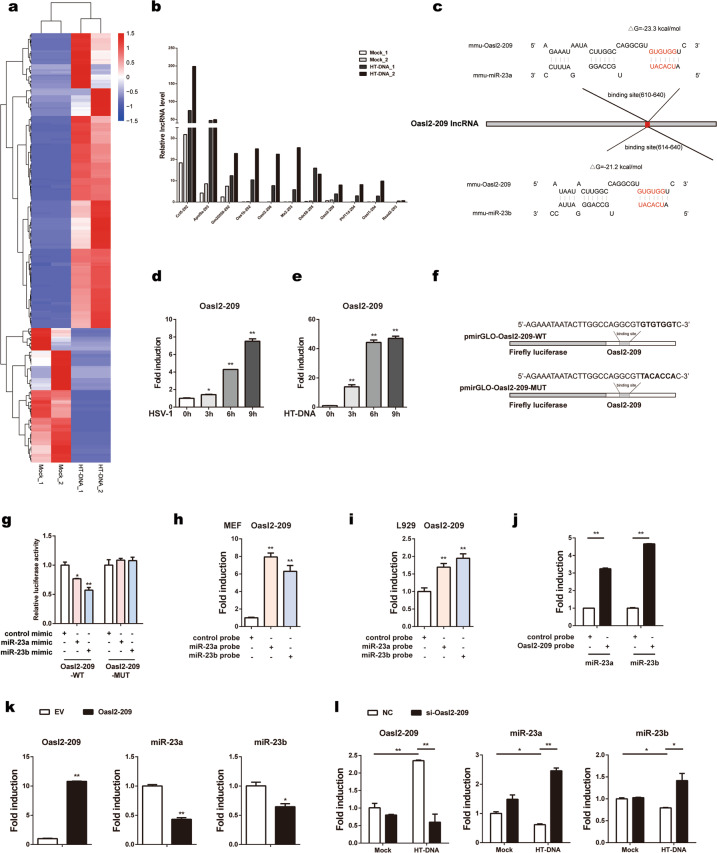
LncRNA Oasl2-209 directly binds to miR-23a/b and regulates miR-23a/b expression. **a** L929 cells were treated with or without HT-DNA (5 µg ml^−1^) for 6 h, and total RNA was then extracted and subjected to high-throughput sequencing. Heat map showing the differentially enriched lncRNAs with adjusted *p* values of less than 0.05 (*padj* < 0.05). **b** qPCR validation of eleven elevated lncRNAs in L929 cells treated as described in **a**. **c** Schematic description of the predicted interactions between the binding site of Oasl2-209 and miR-23a/b. The binding site sequence is marked in red, and the predicted minimum free energy values of the hybridizations are shown. L929 cells were infected with HSV-1 (MOI = 1) (**d**) or stimulated with HT-DNA (5 µg ml^−1^) (**e**) for the indicated times. Then, the level of Oasl2-209 was measured by qPCR. **f** Fragments of Oasl2-209 harboring wild-type (Oasl2-209-WT) or mutant (Oasl2-209-MUT) miR-23a/b target sequences were cloned into pmirGLO reporter plasmids. The mutated nucleotides are marked in bold. **g** HEK293 cells were transfected with miR-23a/b mimics or control mimics along with the indicated reporter plasmids for 24 h. Then, the relative luciferase activities were analyzed. MEFs (**h**) and L929 cells (**i**) were transfected with biotinylated miR-23a/b probes for 48 h and were then harvested for a biotin-avidin pulldown assay. RNA was extracted, and the level of Oasl2-209 was measured by qPCR. **j** L929 cells were harvested and incubated with Oasl2-209 probe-coated streptavidin beads for a pulldown assay. RNA was extracted, and the levels of miR-23a/b were measured by qPCR. **k** L929 cells were transfected with the indicated plasmids, and the levels of Oasl2-209 and miR-23a/b were measured by qPCR. **l** L929 cells transfected with the indicated siRNAs were stimulated with HT-DNA for 6 h, and the levels of Oasl2-209 and miR-23a/b were then measured by qPCR. The data are representative of three independent experiments (mean ± SD). *p < 0.05, **p < 0.01

lncRNAs are a class of transcribed RNA molecules more than 200 nt in length that can modulate miRNA expression/availability by acting as molecular sponges.^31^ In fact, lncRNA-miRNA-mRNA ternary networks have been identified in the regulation of many genes and biological activities.^32^ To explore the underlying mechanisms responsible for miR-23a/b downregulation in response to cytosolic DNA stimulation, we evaluated the potential involvement of lncRNAs by an RNA-seq screen. Briefly, L929 cells were stimulated with HT-DNA (a cytosolic DNA mimic), and total RNA was then extracted and converted into a cDNA library to screen lncRNAs by high-throughput sequencing. The fragments per kilobase of transcript per million mapped reads (FPKM) value was used to represent the abundance of a lncRNA. Among the tested transcripts, the levels of 171 lncRNAs increased, whereas the levels of 78 lncRNAs decreased significantly, with an adjusted *p* value of less than 0.05 (*padj* < 0.05) (Fig. 2a and Supplementary Table 2). Next, we validated the top 18 lncRNAs whose FPKM value was greater than 10, excluding 7 lncRNAs (Micos10-201, Oasl1-203, Nfe2l1-207, Ptprs-221, Dynlrb1-201, A530040E14Rik-203, and Pcbp2-215) that lacked specific primers. As shown by qPCR, the levels of the remaining 11 lncRNAs were elevated significantly upon HT-DNA stimulation (Fig. 2b). Then, the RNAhybrid program was applied to search for lncRNAs that could potentially bind with miR-23a/b. Among the upregulated lncRNAs, only Oasl2-209 was predicted to interact with the seed sequences of miR-23a and miR-23b, with minimum free energy values of −23.3 and −21.2 kcal/mol, respectively (Fig. 2c). Next, we examined the expression dynamics of Oasl2-209 upon cytosolic DNA stimulation. The level of Oasl2-209 in L929 cells increased persistently after stimulation with either HSV-1 (Fig. 2d) or HT-DNA (Fig. 2e). The inverse change trend between the level of Oasl2-209 and those of miR-23a/b suggested that the downregulation of miR-23a/b during dsDNA stimulation might be due to their interaction with Oasl2-209.

To experimentally study whether Oasl2-209 can bind with miR-23a/b, we performed luciferase reporter assays and pulldown assays. We constructed luciferase reporter plasmids for Oasl2-209 (Oasl2-209-WT) and its mutant that lost the ability to interact with miR-23a/b (Oasl2-209-MUT) (Fig. 2f). As shown by the luciferase assay results, overexpression of miR-23a/b suppressed the luciferase activity of Oasl2-209-WT but not Oasl2-209-MUT (Fig. 2g). The biotin-based pulldown assay showed that Oasl2-209 was pulled down in greater amounts by the miR-23a/b probes than by the control probe in both MEFs and L929 cells (Fig. 2h, i). In addition, we used a biotin-labeled probe specific for Oasl2-209 to determine whether Oasl2-209 can pull down miR-23a/b. Consistent with the above results, miR-23a/b were markedly enriched in the precipitates pulled down by the Oasl2-209 probe compared to those pulled down with the control probe (Fig. 2j); these findings collectively confirm that Oasl2-209 can interact directly with the seed sequences of miR-23a/b. Furthermore, we overexpressed and knocked down Oasl2-209 to study its effect on the levels of miR-23a/b (Fig. 2k, l). Overexpression of Oasl2-209 in L929 cells resulted in a decrease in miR-23a/b levels (Fig. 2k). In contrast, knockdown of Oasl2-209 led to an increase in miR-23a/b levels with HT-DNA stimulation (Fig. 2l). Thus, these data indicate that the lncRNA Oasl2-209 can interact with miR-23a/b and downregulate their expression during viral infection.

### miR-23a/b regulate cGAS-mediated type I IFNs production

**Fig. 3:**
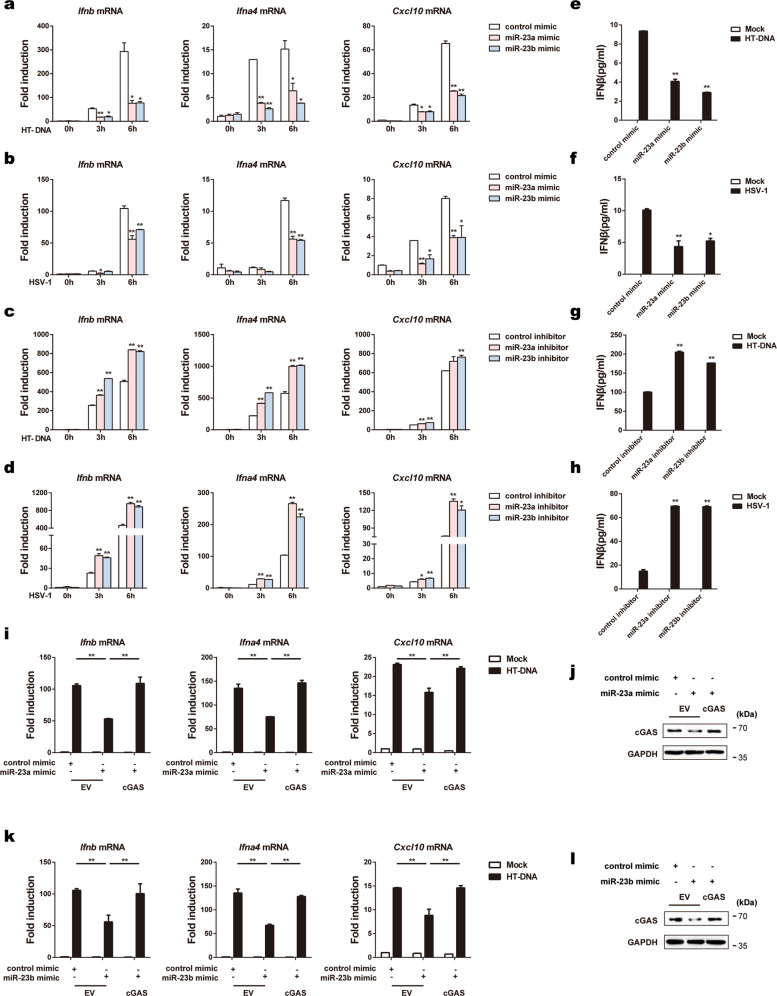
miR-23a/b regulate cGAS-mediated type I IFN production. L929 cells transfected with the indicated mimics were stimulated with HT-DNA (5 µg ml^−1^) (**a**) or HSV-1 (MOI = 1) (**b**) for the indicated times. Induction of *Ifnb*, *Ifna4*, and *Cxcl10* mRNA expression was then measured by qPCR. L929 cells transfected with the indicated inhibitors were stimulated with HT-DNA (5 µg ml^−1^) (**c**) or HSV-1 (MOI = 1) (**d**) for the indicated times. Induction of *Ifnb*, *Ifna4*, and *Cxcl10* mRNA expression was then measured by qPCR. **e**–**h** L929 cells were treated as described in **a**–**d** for 6 h, and the amounts of IFNβ in the supernatants were then determined by ELISA. **i**–**l** MEFs were transfected with the indicated mimics together with the empty vector (EV) or the cGAS expression plasmid (cGAS) and were then stimulated with HT-DNA (5 µg ml^−1^) for 6 h. Then, the mRNA levels of *Ifnb*, *Ifna4*, and *Cxcl10* were analyzed by qPCR (**i, k**), and the protein level of cGAS was examined by western blot analysis (**j**, **l**). The data are representative of three independent experiments (mean ± SD). *p < 0.05, **p < 0.01

Given that miR-23a/b can affect cGAS expression, we then tested whether miR-23a/b regulate cGAS-mediated type I IFN production by transfecting cells with miR-23a/b mimics or inhibitors to increase or decrease their levels, respectively (Supplementary Fig. 2a). As expected, overexpression of miR-23a/b in L929 cells significantly decreased the mRNA levels of IRF3-responsive genes (*Ifnb*, *Ifna4*, and *Cxcl10*) upon exogenous HT-DNA transfection (Fig. 3a) and HSV-1 infection (Fig. 3b). In contrast, knockdown of miR-23a/b increased the expression of the above genes triggered by the same stimulation (Fig. 3c, d). Accordingly, overexpression of miR-23a/b significantly impaired but knockdown of miR-23a/b enhanced the secretion of the IFN-β protein, as measured by enzyme-linked immunosorbent assay (ELISA) (Fig. 3e–h). Importantly, we repeated the transfection experiments in MEFs and obtained similar results (Supplementary Fig. 2b, c), further confirming the contribution of miR-23a/b to cGAS signaling inhibition.

More importantly, compared with transfection with the miR-23a mimic alone, cotransfection of MEFs with the cGAS overexpression plasmid and miR-23a mimic effectively rescued the impairment of cytosolic DNA-induced IRF3-responsive gene expression (Fig. 3i, j). Similarly, restoration of cGAS expression alleviated the inhibitory effects of miR-23b overexpression on the expression of the same antiviral genes (Fig. 3k, l). Taken together, these results suggest that miR-23a/b are able to suppress the production of type I IFNs and inflammatory cytokines by directly modulating cGAS expression.

### miR-23a/b suppress cytosolic DNA-induced cGAS-STING signaling activation

**Fig. 4:**
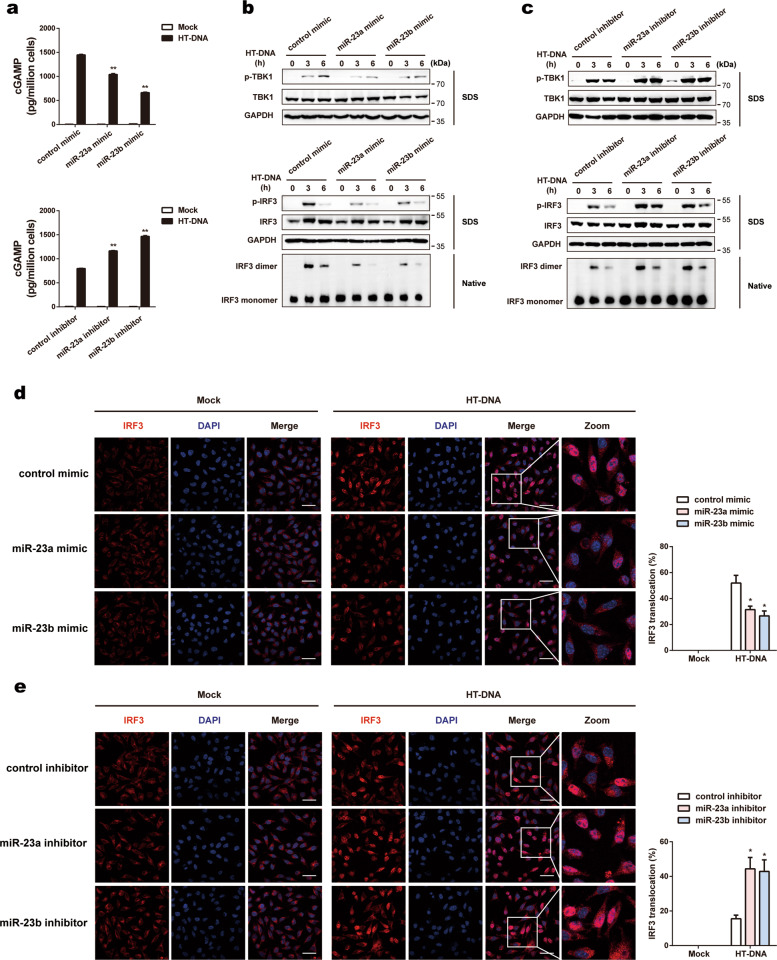
miR-23a/b suppress cytosolic DNA-induced cGAS-STING signaling activation. **a** L929 cells transfected with the indicated mimics (upper panel) or inhibitors (lower panel) were stimulated with HT-DNA (5 µg ml^−1^) for 6 h. Then, the amounts of cGAMP in cell extracts were measured by ELISA. L929 cells transfected with the indicated mimics (**b**) or inhibitors (**c**) were stimulated with HT-DNA (5 µg ml^−1^) for the indicated times. Then, the cell extracts were analyzed for TBK1 and IRF3 phosphorylation by SDS-PAGE or for IRF3 dimerization by native PAGE. L929 cells transfected with the indicated mimics (**d**) or inhibitors (**e**) were stimulated with HT-DNA (5 µg ml^−1^) for 3 h, stained with an antibody against IRF3 (red) and imaged by confocal microscopy. Cells with nuclear IRF3 staining were counted as a percentage of total cells (*n* = 100 cells per sample). The scale bars represent 50 μm. The data are representative of three independent experiments (mean ± SD). *p < 0.05, **p < 0.01

Upon binding to dsDNA, cGAS catalyzes the synthesis of cGAMP, which acts as a second messenger to induce downstream antiviral responses. To study whether miR-23a/b suppresses the activation of cGAS-mediated innate immune signaling, we detected the production of cGAMP in cell lysates by ELISA. As expected, in cells transfected with miR-23a/b mimics, the production of cGAMP was inhibited upon stimulation with HT-DNA, whereas cGAMP production was enhanced in cells transfected with miR-23a/b inhibitors under the same conditions (Fig. 4a).

As TBK1 phosphorylation and IRF3 phosphorylation/dimerization activated by cGAMP are critical for cGAS-mediated signaling responses, we next investigated whether miR-23a/b affects these events. Compared with transfection with the negative control constructs, overexpression of miR-23a/b in L929 cells inhibited the phosphorylation of TBK1 and the phosphorylation/dimerization of IRF3 upon stimulation with HT-DNA (Fig. 4b). In contrast, these processes were markedly enhanced in cells transfected with miR-23a/b inhibitors (Fig. 4c). Substantiating these findings, confocal microscopy revealed that upregulation of miR-23a/b suppressed the nuclear translocation of IRF3 (Fig. 4d), whereas knockdown of miR-23a/b increased the nuclear translocation of IRF3 (Fig. 4e). Collectively, these results suggest that miR-23a/b are essential regulators of cytosolic DNA-induced innate immune responses via targeting of cGAS.

### miR-23a/b promote HSV-1 infection in vitro

**Fig. 5:**
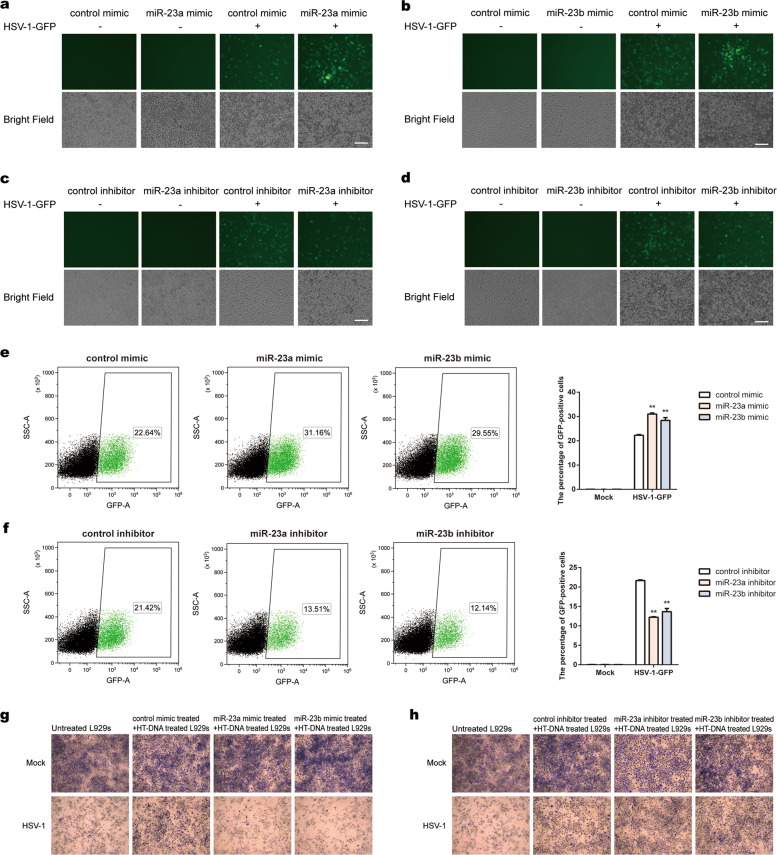
miR-23a/b promote HSV-1 infection in vitro. L929 cells transfected with the indicated mimics (**a**, **b**) or inhibitors (**c**, **d**) were infected with HSV-1-GFP (MOI = 0.3) for 16 h. Then, HSV-1-GFP replication was visualized by fluorescence microscopy. The scale bars represent 100 μm. L929 cells transfected with the indicated mimics (**e**) or inhibitors (**f**) were infected with HSV-1-GFP (MOI = 0.3) for 16 h. Then, the proportions of GFP-positive cells were determined by flow cytometry. L929 cells transfected with the indicated mimics (**g**) or inhibitors (**h**) were stimulated with HT-DNA (5 µg ml^−1^). Fresh L929 cells were incubated overnight with equal volumes of culture supernatants from these treatments and were then infected with HSV-1. Cell proliferation was examined by crystal violet staining. The scale bars represent 250 μm

To investigate whether miR-23a/b facilitate HSV-1 infection, we challenged L929 cells with HSV-1-GFP and measured viral replication. As anticipated, miR-23a/b overexpression led to stronger GFP-positive signals and an increased number of GFP-positive cells (Fig. 5a, b), whereas miR-23a/b inhibition had the opposite effect on viral replication (Fig. 5c, d). Furthermore, we used a FACS-based assay to assess the effects of miR-23a/b on the HSV-1-GFP infection efficiency. Compared with transfection of the control constructs, transfection with miR-23a/b mimics greatly increased the proportion of GFP-positive cells among infected L929 cells (Fig. 5e). In contrast, administration of miR-23a/b inhibitors markedly decreased the proportion of GFP-positive cells (Fig. 5f).

Since IFN-β protects host cells against viral infection, we next collected culture supernatants from L929 cells stimulated with HT-DNA after transfection of miR-23a/b mimics or inhibitors. Fresh L929 cells were incubated with culture supernatants and were then infected with HSV-1. Consistent with the above findings, cells pretreated with culture supernatants from miR-23a/b-overexpressing cells exhibited increased vulnerability to HSV-1 infection (Fig. 5g), whereas knockdown of miR-23a/b promoted host defense against HSV-1 (Fig. 5h). These data suggest that miR-23a/b play important roles in cellular antiviral responses.

### miR-23a/b suppress host defense against HSV-1 infection in vivo

**Fig. 6:**
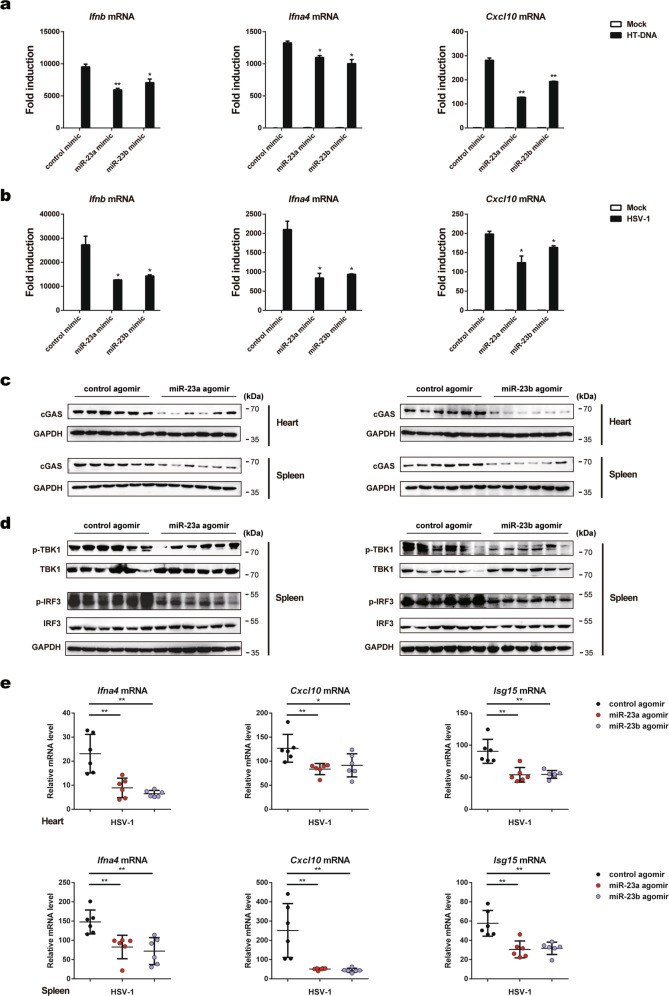
miR-23a/b suppress host defense against HSV-1 infection in vivo. BMDMs from WT mice transfected with the indicated mimics were stimulated with HT-DNA (5 µg ml^−1^) (**a**) or HSV-1 (MOI = 1) (**b**) for 6 h. Then, induction of *Ifnb*, *Ifna4*, and *Cxcl10* mRNA expression was measured by qPCR. **c** WT mice (*n* = 6 per group) were injected intravenously with the indicated agomirs for 48 h and were then infected with HSV-1 (5 × 10^7^ pfu per mouse) for 12 h. Then, the cGAS protein levels in the hearts and spleens were analyzed by western blotting. **d** WT mice (*n* = 6 per group) were treated as described in **c**, and the levels of TBK1/IRF3 phosphorylation in the spleens were then analyzed by western blotting. **e** WT mice (*n* = 6 per group) were treated as described in **c**, and the mRNA levels of *Ifna4*, *Cxcl10*, and *Isg15* in the hearts and spleens were then analyzed by qPCR. The data are representative of three independent experiments (mean ± SD). *p < 0.05, **p < 0.01

To address the in vivo function of miR-23a/b in innate immunity, we transfected bone marrow-derived macrophages (BMDMs) with miR-23a/b mimics. Upregulation of miR-23a/b in BMDMs significantly decreased the expression of *Ifnb, Ifna4*, and *Cxcl10* upon stimulation with HT-DNA or HSV-1 (Fig. 6a, b). Next, we employed a mouse model of HSV-1 infection established by tail vein injection of HSV-1 after treatment (i.v.) with miR-23a/b agomirs or control agomirs (1000 nmol/kg), chemically modified double-stranded miRNA mimics with higher affinity for cell membranes and higher stability for in vivo animal experiments.^33,34^ As illustrated in Fig. 6c, administration of miR-23a/b agomirs markedly reduced the cGAS protein levels in the hearts and spleens. Consistent with the cell-based results, miR-23a/b treatment considerably impaired the phosphorylation of TBK1 and IRF3 in the spleen upon HSV-1 infection (Fig. 6d). Accordingly, the mRNA levels of type I IFNs and ISGs (*Ifna4*, *Cxcl10*, and *Isg15*) were greatly reduced in the hearts and spleens of mice injected with miR-23a/b agomirs compared with control mice (Fig. 6e). Collectively, these data suggest that miR-23a/b suppress host defense against HSV-1 infection by regulating cGAS expression in vivo.

### miR-23a/b suppress cGAS-mediated autoimmunity in mice

**Fig. 7:**
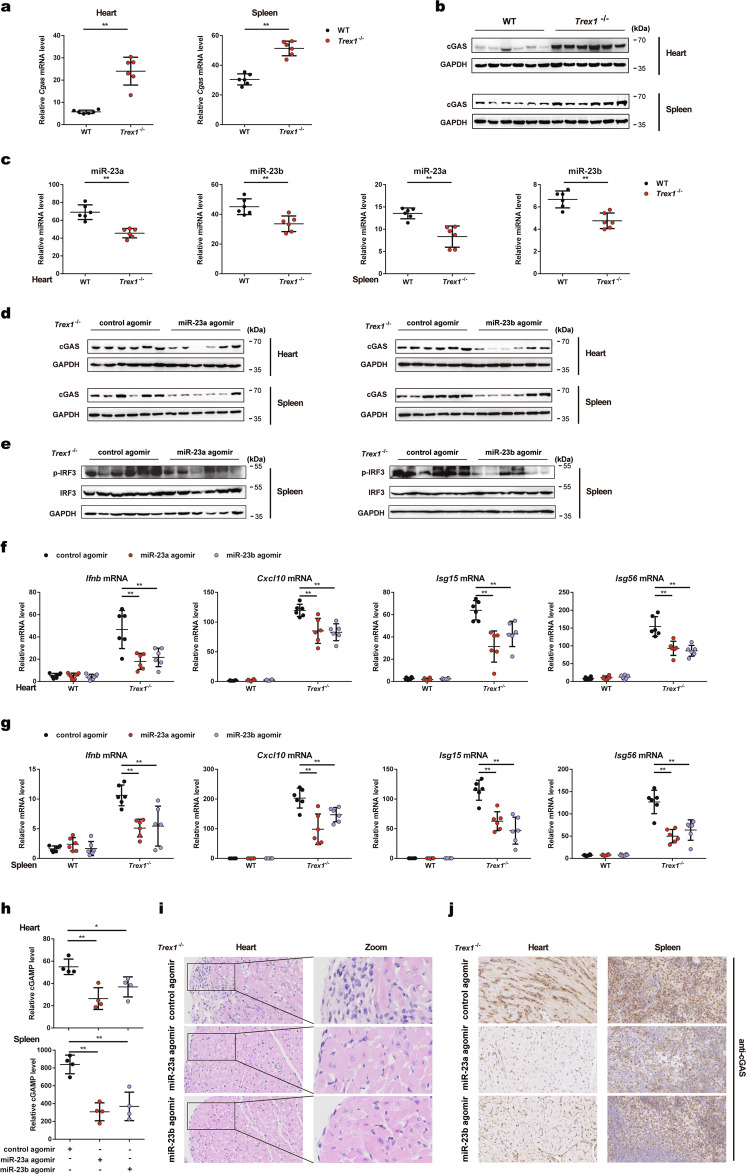
miR-23a/b suppress cGAS-mediated autoimmunity in mice. qPCR analysis of *Cgas* mRNA levels (**a**) and immunoblot analysis of cGAS protein levels (**b**) in the hearts and spleens of WT or *Trex1*^*−/−*^ mice (*n* = 6 per group). **c** qPCR analysis of miR-23a/b levels in the hearts and spleens of WT and *Trex1*^*−/−*^ mice (*n* = 6 per group). **d** *Trex1*^*−/−*^ mice (*n* = 6 per group) were injected intraperitoneally with the indicated agomirs for 6 days, and the cGAS protein levels in the hearts and spleens were then analyzed by western blotting. **e** *Trex1*^*−/−*^ mice (*n* = 6 per group) were treated as described in **d**, and the level of IRF3 phosphorylation in the spleens was then analyzed by western blotting. *Trex1*^*−/−*^ mice (*n* = 6 per group) were treated as described in **d**, and the levels of *Ifnb*, *Cxcl10*, *Isg15*, and *Isg56* mRNA in the hearts (**f**) and spleens (**g**) were then measured by qPCR. **h** *Trex1*^*−/−*^ mice (*n* = 4 per group) were treated as described in **d**, and the amounts of cGAMP in the hearts and spleens were then measured by ELISA. **i** Representative H&E-stained heart sections of *Trex1*^*−/−*^ mice treated as described in **d**. The images in the panels are at ×40 magnification. **j** Representative IHC staining for cGAS in heart and spleen sections of *Trex1*^*−/−*^ mice treated as described in **d**. The images in the panels are at ×40 magnification. The data are representative of three independent experiments (mean ± SD). *p < 0.05, **p < 0.01

As *Trex1*^*−/−*^ mice are mechanistically characterized by aberrant activation of cGAS, we sought to determine whether miR-23a/b could be utilized to treat cGAS-mediated autoimmune diseases. Interestingly, we found that both the protein and mRNA levels of cGAS were significantly elevated in the hearts and spleens of *Trex1*^*−/−*^ mice compared to those of WT mice (Fig. 7a, b). By contrast, the levels of miR-23a/b in the abovementioned tissues were much lower in *Trex1*^*−/−*^ mice (Fig. 7c), consistently revealing an inverse change trend between the cGAS protein level and the miR-23a/b levels. Furthermore, the expression levels of Oasl2-209 were markedly increased in the hearts and spleens of *Trex1*^*−/−*^ mice (Supplementary Fig. 3a), potentially implicating the Oasl2-209-miR-23a/b-cGAS axis in the autoimmune phenotype of *Trex1*^*−/−*^ mice.

To assess the possibility of miR-23a/b for the treatment of cGAS-mediated autoimmunity, we first transfected miR-23a/b mimics or control mimics into BMDMs isolated from WT and *Trex1*^*−/−*^ mice. Overexpression of miR-23a/b inhibited the aberrant upregulation of *Ifnb, Ifna4*, and *Cxcl10* mRNA expression in *Trex1*^*−/−*^ BMDMs (Supplementary Fig. 3b).

Next, we injected *Trex1*^*−/−*^ mice intraperitoneally (i.p.) with miR-23a/b agomirs or control agomirs (500 nmol/kg) daily for 6 days. Administration of miR-23a/b agomirs effectively rescued the miR-23a/b levels in *Trex1*^*−/−*^ mice (Supplementary Fig. 3c, d). As expected, injection of miR-23a/b agomirs effectively inhibited the protein expression of cGAS in the hearts and spleens of *Trex1*^*−/−*^ mice (Fig. 7d). As a result, the levels of IRF3 phosphorylation in the spleens were reduced after administration of miR-23a/b (Fig. 7e). Consistent with the observation that miR-23a/b suppressed cellular cGAS-mediated ISG expression, treatment with miR-23a/b agomirs decreased the expression of type I IFNs and ISGs (*Ifnb, Cxcl10, Isg15*, and *Isg56*) in the hearts and spleens of *Trex1*^*−/−*^ mice (Fig. 7f, g). To determine whether the inhibitory effects of miR-23a/b on *Ifnb* and ISG expression were due to impairment of the cGAS-mediated pathway, we quantified cGAMP production in the hearts and spleens of treated *Trex1*^*−/−*^ mice. Injection of miR-23a/b decreased the cGAMP concentration in both tissues of *Trex1*^*−/−*^ mice (Fig. 7h). H&E staining and cardiac pathology scoring showed that treatment with miR-23a/b agomirs slightly alleviated inflammation in the hearts of *Trex1*^*−/−*^ mice but had no or marginal effects on fibrosis (Fig. 7i and Supplementary Fig. 3e). IHC results revealed obviously decreased cGAS protein levels in the hearts and spleens of miR-23a/b-overexpressing *Trex1*^*−/−*^ mice (Fig. 7j). Taken together, these data indicate that inhibition of cGAS expression by miR-23a/b could be a potential therapeutic strategy for autoimmune diseases.

## Discussion

Recognition of cytosolic foreign DNA by cGAS is critical for triggering an innate immune reaction in the host and for combating infections with various microbes, including DNA viruses, retroviruses, and some bacterial pathogens.^3,35,36^ However, inappropriate chronic activation of cGAS by endogenous DNA contributes to severe autoimmune diseases, such as lupus and AGS.^11,13^ Thus, the activity and expression of cGAS must be tightly regulated to ensure immune homeostasis. Notably, DNA-induced cGAS phase separation, which robustly enhances cGAMP production and innate immune signaling, is critically dependent on the cellular concentration of cGAS, stressing the importance of cGAS expression regulation.^37^ Previously, cGAS expression was shown to be induced in a DNA-triggered IFNβ-dependent manner during pathogen infection^38^; this event was dependent on the existing DNA sensor DDX41 and previous induction of IFNβ. Notably, DDX41 has not been validated as a universal cytosolic dsDNA sensor, and genetic studies using DDX41 knockout mice or human models to validate its role in immune responses to DNA are lacking. In addition, cGAS expression is largely constitutive in most cell types, revealing that it is not regulated at the transcriptional level.^39^ In the present study, we report a novel posttranscriptional regulatory mechanism of cGAS expression via miRNAs. We found that miR-23a/b impaired innate immune responses to cytosolic DNA and DNA viruses by directly targeting cGAS. In addition, miR-23a/b treatment was effective in alleviating cGAS-mediated autoinflammatory responses in the *Trex1*^*−/−*^ mouse model, indicating that miR-23a/b could be used to treat self DNA-induced autoimmune diseases.

To date, numerous miRNAs have been defined as key regulators of different biological processes, particularly those related to innate immunity.^40,41^ However, few miRNAs have been identified to be involved in the regulation of cGAS-STING signaling. miR-29a and miR-378b were reported to activate the cGAS-STING pathway by targeting interferon regulatory factor 7 or binding TANK-binding kinase binding protein 1, respectively.^42^ By targeting NCOA3, miR-25/93 indirectly repressed cGAS expression under hypoxic conditions.^43^ To date, miRNAs with direct effects on cGAS expression have not been reported. We present the first evidence that miR-23a/b can regulate cGAS expression by directly binding with its 3'-UTR. Upon HSV-1 infection, the levels of both miR-23a and miR-23b decreased significantly due to their interaction with the lncRNA Oasl2-209, collectively promoting the elevation of cGAS protein expression and boosting antiviral gene production. A single mature miRNA has the ability to regulate the expression of many genes; thus, it is possible that miR-23a/b suppress type I IFN signaling by targeting other proteins in addition to cGAS. However, several lines of evidence support the present conclusion that the inhibitory effect of miR-23a/b on type I IFN signaling is achieved through specific inhibition of cGAS rather than other molecules. First, knockdown or overexpression of miR-23a/b had no effect on the levels of proteins that are critical for type I IFN production, such as STING, TBK1, IRF3, and MAVS. Second, deficiency of endogenous cGAS induced by miR-23a/b decreased cytosolic DNA-induced production of IFNs and ISGs, which was effectively rescued by exogenous expression of the cGAS plasmid without the cGAS 3'-UTR. Third, miR-23a/b effectively regulated DNA-induced production of 2'3'-cGAMP, which is exclusively produced by activated cGAS.

As cGAS cannot discriminate self from nonself DNA at the structural level, activation of cGAS-STING by self DNA has been proven to be a key event in the initiation and pathogenesis of autoimmune and autoinflammatory diseases. Deletion of cGAS fully rescued the abnormalities in *Trex1*^*−/−*^ mice, underscoring the importance of cGAS inhibition in treating these diseases. Although several cGAS inhibitors have been reported recently, few inhibitors have been tested in autoimmune mouse models in vivo,^44,45^ except for the antimalarial drug derivative X6, which attenuated the autoimmune disease phenotype in *Trex1*^*−/−*^ mice.^30^ Numerous posttranslational modifications have been shown to play important roles in regulating the DNA-sensing ability or enzymatic activity of cGAS,^15,16,46,47^ but chemicals that can specifically/effectively alter these modifications have rarely been identified to date. Moreover, none of these modifications has been linked to cGAS-mediated autoimmunity. Instead, we found that there was an inverse change trend between the levels of miR-23a/b and the cGAS protein level in *Trex1*^*−/−*^ mice. The levels of miR-23a/b were significantly decreased in *Trex1*^*−/−*^ mice, which to some extent contributed to the increase in the cGAS protein level. Accordingly, administration of miR-23a/b significantly decreased the cGAS protein level in *Trex1*^*−/−*^ mice. However, treatment with miR-23a/b only slightly ameliorated the inflammatory phenotypes and had little or no effect on heart fibrosis in *Trex1*^*−/−*^ mice. A longer period of treatment or treatment of the pups as soon as possible after birth might be useful to improve the outcomes. However, miR-23a/b administration markedly decreased self DNA-induced *Ifnb* and ISG expression in the tissues of *Trex1*^*−/−*^ mice. Thus, our research identifies a feasible approach to treat cGAS-mediated autoimmune diseases.

In addition to mediating autoimmune diseases, deregulation of cGAS is associated with other pathological processes, such as tumor and cellular senescence.^48–51^ miR-23a has been reported to be upregulated during skin aging and to accelerate senescence, whereas the potential molecular mechanisms of miR-23a/b in the initiation and progression of cellular senescence remain largely unknown.^52,53^ Thus, future studies are needed to delineate whether miR-23a/b contribute to other cGAS-related diseases and determine the effectiveness of miR-23a/b for the treatment of these diseases.

## Supplementary information

Marked-up Supplementary material

Non-highlighted Supplementary material
