# Supplementary material for: miR-23a/b suppress cGAS-mediated innate and autoimmunity
Source: Cell Mol Immunol. 2021 Mar 25;18(5):1235–48. doi: 10.1038/s41423-021-00668-x (PMC8093233; doi:10.1038/s41423-021-00668-x)
Supplement: Supplementary file 1 — Marked-up Supplementary material [file 41423_2021_668_MOESM1_ESM.docx]

**Supplementary information**

**
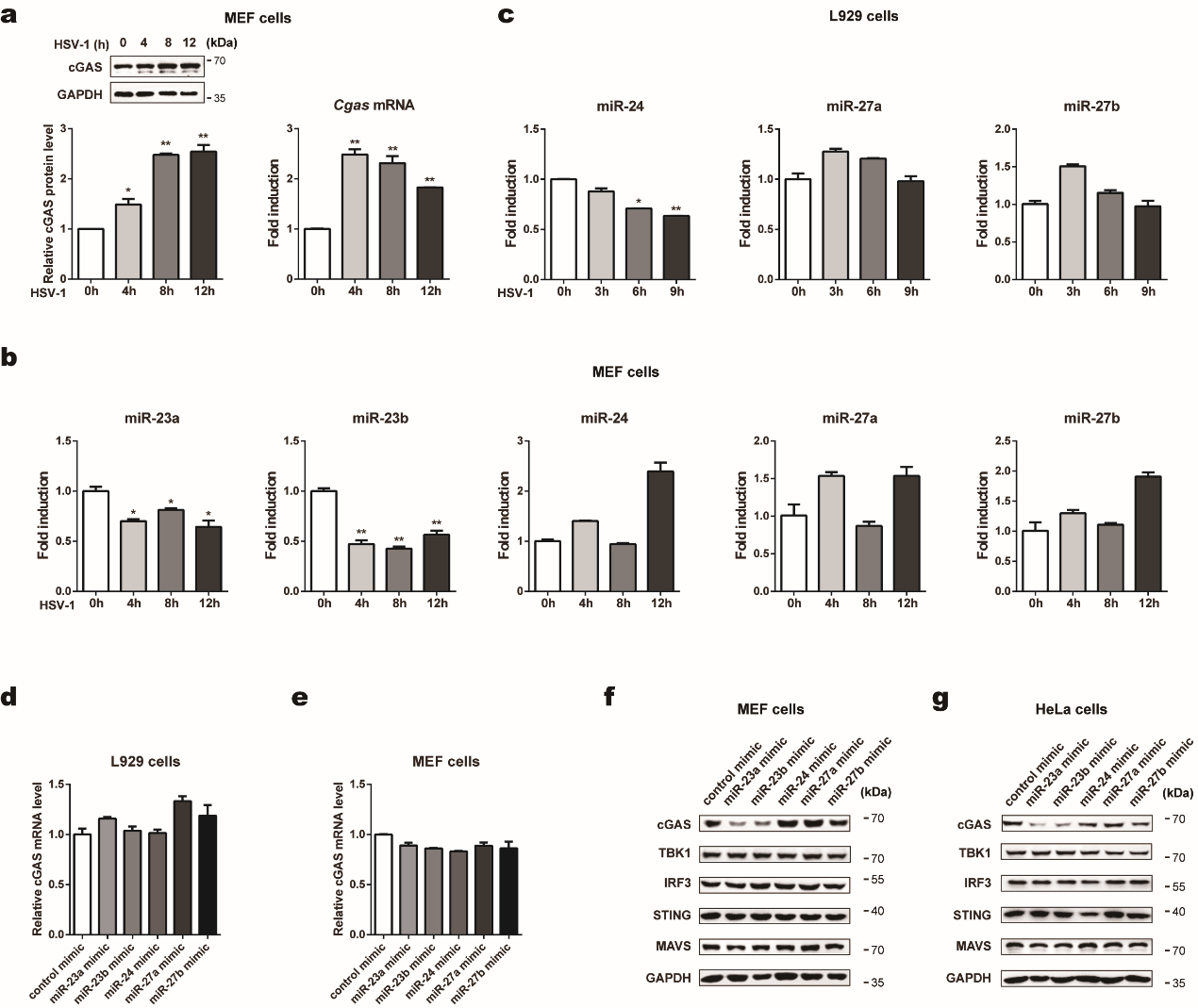
**

**Supplementary Figure 1. miR-23a/b directly regulate cGAS expression. (a)** MEF cells were infected with HSV-1 (MOI = 1) for the indicated times. Then the cGAS protein levels were analyzed by western blot and the relative band intensity was quantified by Image J (left panel), and the cGAS mRNA levels were measured by qPCR (right panel). **(b)** MEF cells were infected with HSV-1 (MOI = 1) for the indicated times, then the levels of miR-23a, miR-23b, miR-24, miR-27a and miR-27b were measured by qPCR. **(c)** L929 cells were infected with HSV-1 (MOI = 1) for the indicated times, then the levels of miR-24, miR-27a and miR-27b were measured by qPCR. **(d, e)** L929 cells (**d**) or MEF cells (**e**) were transfected with the indicated mimics, then the cGAS mRNA levels were analyzed by qPCR. **(f, g)** MEF cells (**f**) or HeLa cells (**g**) were transfected with the indicated mimics, then the protein levels of cGAS, TBK1, IRF3, STING and MAVS were analyzed by western blot. Data are representative of three independent experiments (mean ± SD). * *p* < 0.05, ** *p* < 0.01.


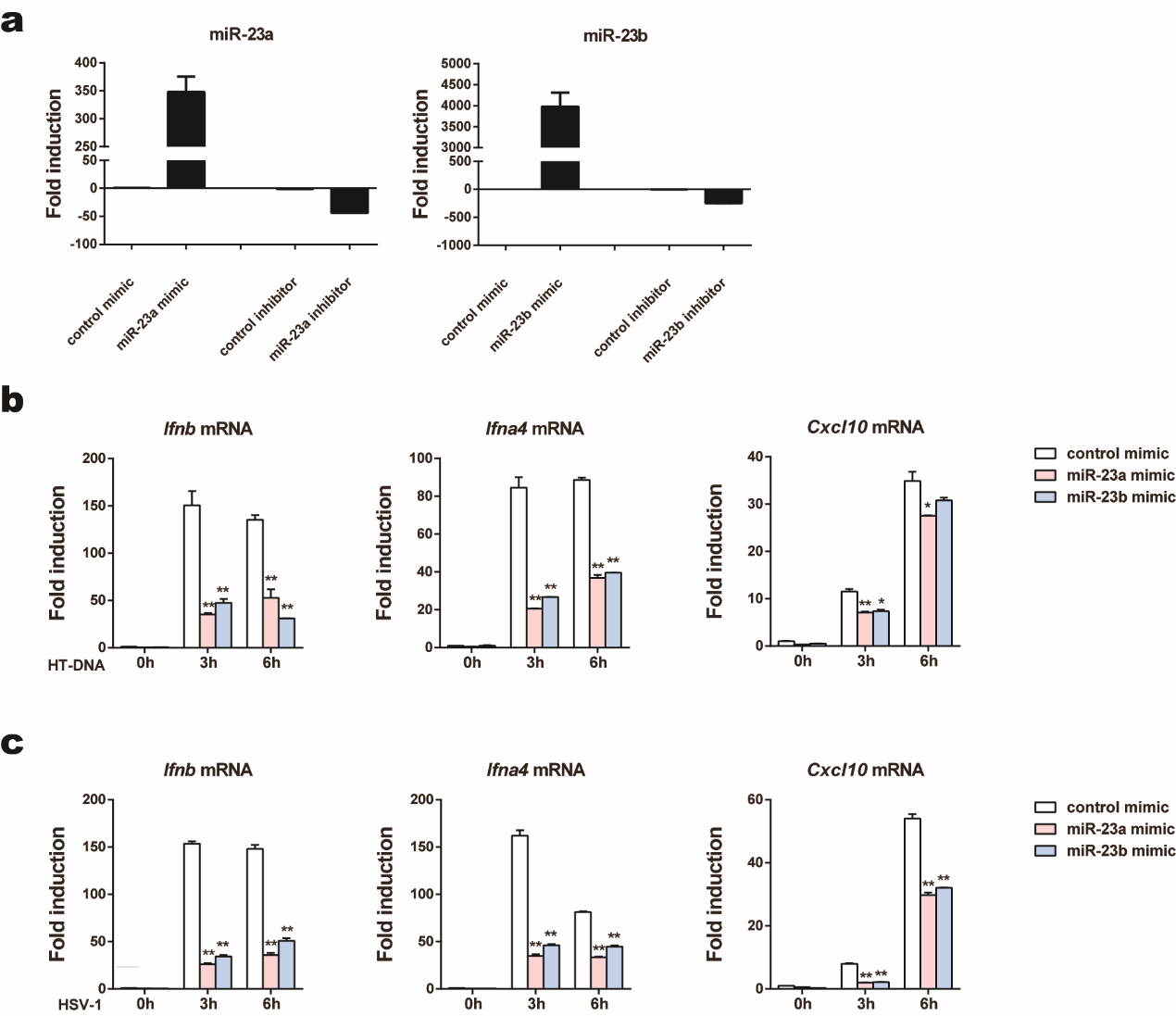


**Supplementary Figure 2. miR-23a/b regulate cGAS-mediated type I IFNs production. (a)** L929 cells were transfected with the indicated mimics or inhibitors, then the levels of miR-23a/b were examined by qPCR. **(b, c)** MEF cells transfected with the indicated mimics were stimulated with HT-DNA (5 ug•ml^-1^) (**b**), or HSV-1 (MOI = 1) (**c**) for the indicated times. Then the mRNA levels of *Ifnb*, *Ifna4* and *Cxcl10* were measured by qPCR. Data are representative of three independent experiments (mean ± SD). * *p* < 0.05, ** *p* < 0.01.


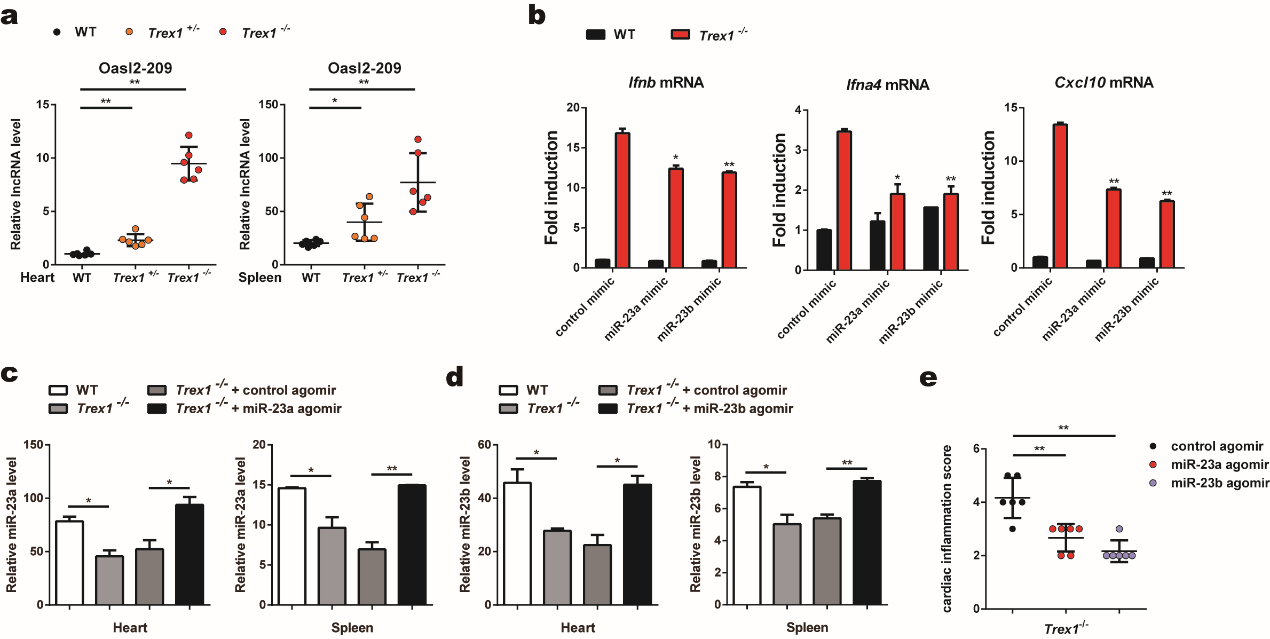


**Supplementary Figure 3. miR-23a/b suppress cGAS-mediated autoimmunity in mice.** **(a)** The levels of Oasl2-209 in the hearts and spleens of WT, *Trex1^+/-^* or *Trex1^-/-^* mice (n = 6 per group) were measured by qPCR. **(b)** BMDMs from WT or *Trex1^-/-^* mice were transfected with the indicated mimics, then the induction of *Ifnb*, *Ifna4* and *Cxcl10* mRNA expression was measured by qPCR. **(c, d)** *Trex1^-/-^* mice were injected intraperitoneally with the indicated agomirs for 6 days. The levels of miR-23a (**c**) and miR-23b (**d**) were then analyzed by qPCR. **(e)** Pathological scores of cardiac inflammations in *Trex1^-/-^* mice administrated with the indicated agomirs for 6 days. Data are representative of three independent experiments (mean ± SD). * *p* < 0.05, ** *p* < 0.01.

**Supplementary Table 1. The primers in RT-qPCR assays.**

| Primers | Sequence (5' - 3') |
| --- | --- |
| *Gapdh*-sense | GAA GGG CTC ATG ACC ACA GT |
| *Gapdh*-antisense | GGA TGC AGG GAT GAT GTT CT |
| *Cgas*-sense | GTC GGA GTT CAA AGG TGT GGA |
| *Cgas*-antisense | GAC TCA GCG GAT TTC CTC GTG |
| *Ifnb*-sense | AGA TCA ACC TCA CCT ACA GG |
| *Ifnb*-antisense | TCA GAA ACA CTG TCT GCT GG |
| *Ifna4*-sense | ACC CAC AGC CCA GAG AGT GAC C |
| *Ifna4*-antisense | AGG CCC TCT TGT TCC CGA GGT |
| *Cxcl10*-sense | CGA TGA CGG GCC AGT GAG AAT G |
| *Cxcl10*-antisense | TCA ACA CGT GGG CAG GAT AGG CT |
| *Isg15*-sense | GGA ACG AAA GGG GCC ACA GCA |
| *Isg15*-antisense | CCT CCA TGG GCC TTC CCT CGA |
| *Isg56*-sense | AGT GCA GGC AGA AAT TCA CC |
| *Isg56*-antisense | AGC AGT CAG TAG TTT CCT CC |
| Oasl2-209-sense | TGT GCC TTT GCT TCC TGT CT |
| Oasl2-209-antisense | ATA TCA GGC TGG CCT CGA AC |
| Ccl5-202-sense | TTC TCA ATC TGT GGG TCG GG |
| Ccl5-202-antisense | TCT CAT GTT GTG GTG ACC CC |
| Apol9a-203-sense | AAG GGA GAT GAA GTG GGG GA |
| Apol9a-203-antisense | GAA GAA GGT TGC TGA GGC CT |
| Gm20559-202-sense | GCA GAA GCT GGA AAG GTT GC |
| Gm20559-202-antisense | GAA AGT TGC CCG TCT CTG GA |
| Oas1b-202-sense | ATG AGG GCC TCT AAA GGG GT |
| Oas1b-202-antisense | ATC CAG TCA CAT AGC AGG CG |
| Oasl2-208-sense | GTG CAT GTG TGT GAG GGG TA |
| Oasl2-208-antisense | GGT GTT GCC AAA GTC ACG TC |
| Mx2-203-sense | GGG ACG TAC CTT CAT AGC CA |
| Mx2-203-antisense | CAC ATG AAC ACA CGC ACA CA |
| Ddx58-205-sense | CCT GAC GTG GCT TGT TGT TG |
| Ddx58-205-antisense | TGG TGA TGC AGG CCT TCA AT |
| Phf11d-204-sense | GTC TGT TCT CTG CAA CGG GA |
| Phf11d-204-antisense | CAA CAC TGG AAG CCA ACA GC |
| Oasl1-204-sense | ACC TCA GAC CCT GAC ACT GA |
| Oasl1-204-antisense | CAC TGG AGT GGT TTA GGG CA |
| Rsad2-203-sense | AGG ACC ACT CAG CAG GAT CT |
| Rsad2-203-antisense | TCT CAC AAG CTT GCC CAA GT |

**Supplementary Table 2. RNA-seq result of the top 18 upregulated lncRNAs in L929 cells stimulated with HT-DNA.**

| Transcript id | transcript  name | HTD  FPKM | MOCK  FPKM | log2FoldChange | | padj | |
| --- | --- | --- | --- | --- | --- | --- | --- |
| ENSMUST00000063040 | Micos10-201 | 54.83 | 1.669 | | 5.151 | | 4.92E-02 |
| ENSMUST00000125015 | Ccl5-202 | 42.47 | 0.000 | | 14.725 | | 5.42E-05 |
| ENSMUST00000137903 | Ddx58-205 | 37.18 | 0.702 | | 5.922 | | 1.12E-02 |
| ENSMUST00000182725 | Oas1b-202 | 28.47 | 0.045 | | 9.494 | | 6.10E-03 |
| ENSMUST00000140159 | Oasl1-203 | 28.33 | 0.000 | | 16.991 | | 9.29E-08 |
| ENSMUST00000201831 | Gm20559-202 | 26.97 | 0.921 | | 5.011 | | 3.65E-02 |
| ENSMUST00000201445 | Oasl2-208 | 24.04 | 0.039 | | 9.248 | | 2.06E-03 |
| ENSMUST00000161754 | Phf11d-204 | 21.71 | 0.121 | | 7.694 | | 8.62E-04 |
| ENSMUST00000190097 | Mx2-203 | 21.54 | 0.050 | | 8.575 | | 3.09E-02 |
| ENSMUST00000229175 | Apol9a-203 | 20.87 | 0.400 | | 5.868 | | 3.46E-02 |
| ENSMUST00000135836 | Nfe2l1-207 | 18.60 | 0.171 | | 6.857 | | 3.14E-02 |
| ENSMUST00000225785 | Ptprs-221 | 16.27 | 0.000 | | 15.738 | | 2.22E-02 |
| ENSMUST00000054912 | Dynlrb1-201 | 15.82 | 0.001 | | 14.068 | | 1.75E-04 |
| ENSMUST00000152329 | Oasl1-204 | 14.82 | 0.144 | | 6.769 | | 2.96E-02 |
| ENSMUST00000161150 | A530040E14Rik-203 | 14.42 | 0.028 | | 9.196 | | 1.41E-02 |
| ENSMUST00000142732 | Rsad2-203 | 14.17 | 0.000 | | 14.385 | | 1.53E-04 |
| ENSMUST00000229822 | Pcbp2-215 | 13.61 | 0.117 | | 7.185 | | 4.87E-02 |
| ENSMUST00000201919 | Oasl2-209 | 10.41 | 0.008 | | 8.423 | | 2.81E-02 |
| ENSMUST00000135184 | Mx1-203 | 9.36 | 0.000 | | 15.817 | | 1.55E-06 |
